# Supplementary material for: Behavioural activation activities for employees in the Chinese culture: A workshop
Source: PLOS Ment Health. 2024 Dec 20;1(7):e0000190. doi: 10.1371/journal.pmen.0000190 (PMC12798598; doi:10.1371/journal.pmen.0000190)

First, please fill this questionnaire:
https://www.wjx.cn/vj/h3kys56.aspx#
Pick your fav colour below and fill your details (do not use real names)

Example:

Name：小绒鼠
Age：2
Job：主播

Male 1-4 yr working exp

Male 5+ yr working exp

Female 1-4 yr working exp

Female 5+ yr working exp

Provide details

debate

名称：
年龄：
工作：

名称：
年龄：
工作：

名称：
年龄：
工作：

名称：
年龄：
工作：

名称：
年龄：
工作：

名称：
年龄：
工作：

行为激活(Behavioral activation, BA)

Below are some activities suggested by western research that could have positive impact on mental wellbeing. As a Chinese Employee, please read these activities.
Mark the activities you think will be beneficial as ★, mark ✸ that are not. Add new activities with sticker ■ .

e.g. :

Your marks:↓

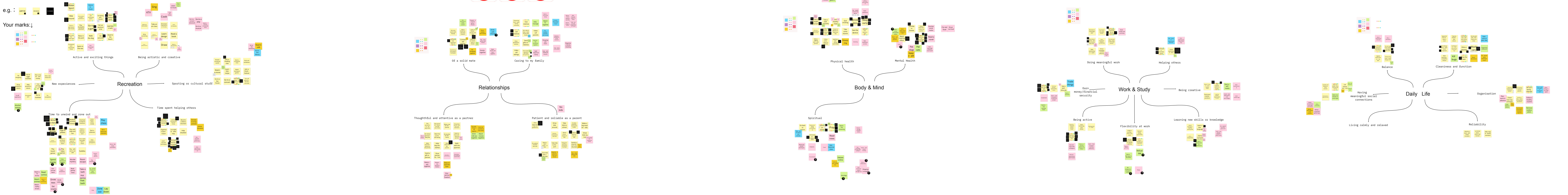

Supplement: S2 File — (PDF) [file pmen.0000190.s003.pdf]
