# Supplementary material for: Behavioural activation activities for employees in the Chinese culture: A workshop
Source: PLOS Ment Health. 2024 Dec 20;1(7):e0000190. doi: 10.1371/journal.pmen.0000190 (PMC12798598; doi:10.1371/journal.pmen.0000190)
Supplement: Attachment — Submitted filename: Response to Reviewers.docx [file pmen.0000190.s005.docx]

Reviewer #1:

**Overall:** Please include page and line numbers. It is also important to justify (distribute text evenly between margins)
Title “Behavioural activation activities for Chinese employees: a workshop” why not behavioral activation therapy for Chinese employees to reflect the exact intervention under consideration. The aim of the study is also not well reflected in the title.
Abstract: Include information of method of data collection and analysis.
Background: Background is well written, although it relies most on the comparison between Australia and China, yet the title does not portray the study as a comparative study

**2.1** Objectives and overview: since the study approach is mentioned under this section, please provide a justification for the choice of approach. Information about participants recruitment should be moved to 2.2.2 ‘participants’
It is also unclear to me if this was a mixed methods study. From the text, it is difficult to understand but looking at figure 1, it looks like one. Offer clarity on this, and if it’s a mixed methods study, mention the exact type of MM used and a justification for the choice.

**2.2** Workshop: Adapting digital BAT is a bit different from adapting a face-to-face BAT. Looks like the authors were interested in digital BAT but this does not come out well in the background.

**2.2.1** ‘Participants’. Give additional details about the recruitment process. How did the potential participants get to know about this study? How was the consent process handled?

#Participant’s demographics should be moved to the results section and put in a table

**2.2.2** Materials and settings: Information presented under these sections is basically about ‘procedure’. The contact and the title contradict.

#Can the authors include a table summarizing the workshop contents? Clearly indicating the number of workshops and the duration the workshops lasted? What was the criteria of assigning participants to different workshops?

**2.2.3** Protocol: It remains unclear to me whether all participants were attending one online workshop at a time or they were in separate workshops. In case of the latter, what was the consideration for assigning participants to the separate workshops? Who facilitated the discussions? Where these recorded? Earlier, the authors mention that the workshops varied from 1.5 -3 hours, what caused the variation in time? did this time cover the overall presentation, presentation of perspectives and discussing possible modifications?

**2.3.1**. Data collection: Experience of living abroad first appears under this section, any justification?
\# I have completely failed to understand the method of data collection used to collect participants proposals on new activities.
#Was there a data collection guide used during the workshops? If Yes, can sample questions be included here and the guide provided as supplementary material?

**2.3.2** Data analysis: I have many questions about this section, particularly the qualitative part. Earlier alone the authors indicated that the study was guided by Hofstede’s framework, I have not seen that guide data analysis. Second, if the study was guided by a framework, how can data be analyzed inductively? What was the role of the framework? The data analysis process is also not detailed and robust, it is difficult to understand how data was delt with. More details are needed.

Were the workshops recorded? And then data transcribed? If so, who translated the data to English? Were participants involved in the translation to avoid misrepresentation?
\# The robustness of qualitative analysis is found in the details of how the data was handled. This information is not provided anywhere. Who analyzed the data? What was their level of qualifications?

\# Given that there was a lot of qualitative data collected, a statement of trustworthiness needs to be included. Secondly how was social desirability delt with?

\# Since the end goal was identifying elements of the BAT for cultural and contextual adaptation, using an adaptation framework would have helped the study. The study seems to have been targeted at assessing social validity of BAT and if this was the case, did participants have sufficient knowledge about the BAT to be able to provide useful feedback beyond liking and not liking? In the composition of participants where mental health experts (who blend both professional knowledge and understanding of the context) involved?

*The title was changed to "Behavioural activation activities for employees in the Chinese culture: a workshop " to better reflect the cross-cultural nature of the study and its purpose.*

*Thank you very much for your comments for the methodology section. The following revisions have been made based on your feedback.*

1. *Demographics were elaborated including participates’ age, educational level, gender and living abroad experience, with charts. Demographics is moved to the beginning of the results section.*
2. *The approach of the study is further explained. It is a mixed-methods study combined quantitative methods, where participants' votes on each activity on the Miro board were recorded and statistically analyzed, with qualitative methods, capturing and thematically analyzing conversation content. This method was chosen because it facilitates an inclusive, collaborative environment that harnesses the diverse perspectives of participants, essential for culturally sensitive adaptations.*
3. *Recruitment details explained. Participant recruitment was advertised on Mandarin Chinese social messaging platforms as posts, encouraging people with interests to contact the researchers or repost to potential subjects. No financial compensation was given. Participants were recruited online from 1st September 2022 to 15th September 2022. The dissemination of the contact link was stopped after 50 participants were recruited, whose age ranged from working experience ranged from 1 year to 30 years, reflecting a diverse range of professional backgrounds.*
4. *The “Materials and settings” part was renamed as “procedure” and combined with the following “protocol” part. It is made clear that participants were allocated into 12 workshops based on their time preference and educational levels to ensure they can understand each other effectively. Each participant only participated in one workshop. Workshop structure and activities included in the workshop will be submitted as appendix.*
5. *The inclusion of living abroad as a variable was justified by the hypothesis that participants' exposure to Western cultures could potentially explain their understanding of and reactions to the Western cultural influences behind the design of the original activities.*
6. *The collection of data was further elaborated: The "star" and "cross" markings provided by the participants on the online whiteboard were recorded and subjected to statistical analysis with respect to participants' gender, working history, educational level, and experience of living abroad. Furthermore, all the new activities proposed by the participants on the whiteboard were recorded and later categorized according to their corresponding themes during the analysis. The discussion audios of the workshops were recorded and transcribed by a bilingual researcher after the workshop. he markings of the 12 workshops were aggregated and statistically analysed to identify the controversial activities. For the qualitative analysis, an inductive thematic analysis of the transcribed text was conducted by a bilingual designer with psychology background and a bilingual psychologist using NVivo.*
7. *It is made clear that the participants were introduced to BAT at the beginning of the workshops.*

**3.** Results: Better to start this section with reporting participants demographics
#Controversial activities” was this the main aim of the study? You seem to be giving it too much importance.

**3.1.** “These” Controversial Activities” were observed at two distinct layers. Firstly, the results conveyed culture-specific messages that diverged from the corresponding findings in Australia”
Was this a comparative study? If yes, then it should be reflected in the title and background.

#If the study was guided by Hofstede’s framework, it should then guide presentation of results and the discussion.

**3.1.1** Sports: Participant voices are not represented in this section of results. Adding quotes would have improved trustworthiness of the results
\# The arrangement of the manuscript is a bit confusing. Looks like the discussion starts right away from presentation of results. I suggest the results section is only left for results.
**3.3** Mental health experts “Unlike Australia, the mental health system is highly developed, well-established, and widely accessible, despite the gravity of the mental health issue for Chinese employee, the regulation of the mental health industry in China remains insufficient” Is this a statement made by participants? Given that this is a results section, could the authors just leave it to results?
\# This statement could be revised to bring out the intended meaning.

\# citing the immaturity of psychiatric and counselling services in China due to their inaccessibility and high costs. Some participants with prior experience with mental health professionals expressed concerns about their expertise, thereby worrying about the potential risks of exacerbating their conditions through misdiagnosis” Is this a finding from the current study? I suggest the authors resist from speaking beyond the results.

Table 3: The second category in Table 3 is misleading “English Name (abbreviated, what exactly are the authors communicating?
#Table 2 and 3 have different formats, can the authors use one format throughout the manuscript?

**3.5** Summary of findings: I recommend it is removed because it is a repetition of the introduction part of the results.

**4.1** Discussion. The objective of the study listed under the discussion differs from the objective set earlier.

\# Under 4.1 authors talk about the intention to conduct further analysis using Hofstede’s cultural dimensions and immediately start presenting results following the dimensions in 4.1.1. when was the analysis mentioned above done to be able to guide the following discussion?

#If the authors wanted Hofstede’s dimensions to guide the discussion, they should have used the dimensions to guide data collection and presentation of results. There are so many things mixed up.
\# The discussion needs to be improved to clearly reflect the study results.

**4.2** Limitations. Authors list small sample size as a potential cause of bias. If the study methods were clear, this could have been handled well. 46 participants in a qualitative study cannot be considered to be a small size, however, since they study also talks about quantitative data somewhere, this is where the confusion started from.

*For the results and discussion sections, the below changes were made based on the suggestions*

1. *Further clarification was provided regarding the role of the controversial activities in the study. The importance of the “controversial activities” are related to the cross-culture nature of this study. Our research method is based on a similar study conducted in Australia - the BAT activities we used in our study was co-designed by Australian psychologists and employees which was mentioned in the paragraph. Our objective is to identify positive activities among participants in Chinese culture.*
2. *The role of Hofstede's framework in this research has been elucidated. The framework is employed exclusively in the discussion section, serving as a cultural lens to guide our examination of potential socio-cultural origins that may influence the results. We have, nevertheless, explicitly stated that this framework does not guide our research design.*
3. *A participant's quotation has been incorporated into the sports section, and we have delineated a clear separation between the results and discussion sections.*
4. *All content beyond the empirical findings in the results section has been excised, including commentary regarding China's mental health system and services. The results section now presents only the direct findings from the study.*
5. *The summary of findings has been removed from the study to avoid redundancy.*
6. *The objective in the discussion section has been revised to: 'The objective of this study is to identify "positive activities" suitable for behavioral activation therapy within the cultural context of China.' This revision ensures consistency with the objective established earlier in the paper.*
7. *As previously mentioned, the role of Hofstede's dimensions has been clarified, and the results analysis and discussion have been segregated to prevent confusion.*

Reviewer #2:

Thank you for the opportunity to read and review this article.
I have a few comments below.

Introduction
1 What is a "more positive behaviours"? It seems that there are less positive behaviours.
2. The authors have made an effort to justify the topic and emphasise the relevance of the study. I believe that the reader should have a clearer definition of the terms.
3. I don't see the need for table 1.

Methods
1. The title 2.1. is unclear. It says that it is about the aim of the study, but nothing is said about the aim. In fact, the objective has already been presented, so the title is not appropriate.
2. What was the criterion for selecting the participants? Where in China are the participants from?
3. In point 2.2.2. it's not clear what the materials are.
4. Figure 2 is not clear.
5. What statistical analysis was carried out?

Results
1. The abbreviations in table 2 are not understood (e.g. NE, DF).
2. What statistical treatment was carried out?
3. It is not clear why the results observed are often compared with data from other studies with Australians.

*"More positive behaviors" has been changed to "mood-enhancing behaviors" to avoid confusion and facilitate a better understanding of the term. Table 1 has been removed.*

*The title of 2.1 is changed to “overview” as suggested. The selection of participants were explained. Participant recruitment was advertised on Mandarin Chinese social messaging platforms as posts, encouraging people with interests to contact the researchers or repost to potential subjects. No financial compensation was given. Participants were recruited online from 1^st^ September 2022 to 15^th^ September 2022. The dissemination of the contact link was stopped after 50 participants were recruited, whose working experience ranged from 1 year to 30 years, reflecting a diverse range of professional backgrounds.*

*Demographics details were included in the beginning of the results section. The statistics abbreviation of NE and DF is further explained to better reflect the statistical method. 2.2.2 was rewritten “procedure” and the activities presented to the participants will be submitted as appendix. Figure 2 was updated with English translation and explanation.*

*The study was based made as a comparison with the Australian study of Headgear. The BAT activities originally presented to the participants were co-designed by Australian psychologists and employees. The connection between this study and the Australian study was further explained in the title and introduction section.*

*In the limitation section, the format of the workshop as being online was further discussed.*

Reviewer #3:

The study presents a thorough examination of the workshop's design, materials, and protocol, offering a transparent portrayal of the methodology. Additionally, the researchers conducted an exhaustive analysis of the data, encompassing both quantitative assessment of participants' engagement with activities and qualitative examination of discussions. However, certain crucial aspects necessitate further elaboration:
1. Inclusion and Exclusion Criteria: The methods section should provide explicit details regarding the criteria used for participant selection, including any exclusion criteria employed. The authors should clearly specify whether individuals with pre-existing mental health conditions, such as depression or anxiety, were excluded from the study.
2. Participant Demographics: The authors need to provide more detailed information on demographic data of partcipants such as age, gender,etc. Also, the participants' work variables, such as type of occupation and working hours. Additionally, the relationship between participant demographics, such as gender, and the study's aims should be discussed.
3. Response Rate and Decline Rate: Given the online nature of the study, it is imperative for the authors to disclose the response rate, indicating the proportion of invitations accepted, as well as the decline rate. These statistics offer insights into the sample representativeness and potential biases.
4. Dropout Rate: Throughout the duration of the research, tracking the dropout rate is essential. This provides an understanding of participant attrition and its potential impact on the study's outcomes.
5. Limitations: The authors should acknowledge the limitations of the online workshop format, which may have limited the depth of interactions and discussions compared to in-person sessions.

*Thank you very much for your comments.*

*The participants selection criteria are further explained. Eligible participants were required to be Chinese nationals, currently employed or previously employed, and have access to computers and the internet. The mental well-being levels of the participants were not assessed and did not serve as a criterion for their selection. This decision was made because we did not expect the discussions around BAT activities in the workshops to evoke negative effects on their potential mental ill-being.*

*Demographics data were included at the beginning of the results section with charts.*

*Unfortunately, as participant recruitment was advertised on Mandarin Chinese social messaging platforms as posts, encouraging people with interests to contact the researchers or repost to potential subjects, the response rate and decline rate was unknown. Dropout Rate was included: Throughout the research, 4 (8%) of the 50 recruited participants dropped out before the workshop started. Another two (4%) participants left the workshop midway due to urgent work obligations, with their statements prior to leave were still recorded for qualitative analysis with their consent.. The rest 44 (88%) of them completed the workshop tasks to the end.*

Reviewer #4:

Thank you to the authors for studying an underreported topic in behavioral activation activities within a Chinese cohort; highlighting regional differences from previous research. Though the topic is well founded, there are a few recommendations to help amplify the quality of the manuscript.

Methods: Though attached separately; a sentence specifying accordance with STROBE guidelines should be included in the methods of the manuscript. Additionally, further elaboration regarding the participants of the study if possible, by specifying whether any participants were present at >1 workshop; and whether any compensation was given would help improve transparency. Lastly, please specify whether the 201 activities included from the Headgear app were the total number present on the application, or a subset. If a subset of activities were selected for the study, the reason for such selection would be needed.

Results: Please specify demographics data that was mentioned in the methods in the results, as that would allow for further consideration of potential skew impacting the results (a younger population within the study may be inclined to select certain activities). Additionally, this study utilizes the results section to compare data with data from the previous study on an Australian cohort. Such comparisons should be restricted to the discussion section of the manuscript.

Discussion: The discussion brings good points to compare the pre-existing dynamics within China and it's impact on activities preference. However, at times such points are not supported with references, such as the discussion on workplace culture. If possible, please add supporting references from literature to further solidify the points made by the discussion.

Overall, this paper presents a novel idea with the implementation of a good study design to collect qualitative data. Additionally, the rationale for the implementation is well founded and expanded upon in the introduction, with the discussion making good points regarding the disparities noted in contrast to previous studies.

*Thank you very much for your comments.*

*A sentence specifying accordance with STROBE guidelines was included in the methods of the manuscript.*

*The participant’s part is further elaborated: No financial compensation was given. Each participant only participated in one workshop. The 201 activities included from the Headgear app were the total number present on the application.*

*Demographics data were included at the beginning of the results section with charts.*

*All content extraneous to the empirical findings was excised from the results section, including any comparative analysis with the Australian study. This adjustment ensures that the results section remains purely data-driven, presenting only the direct, objective outcomes from our Chinese participant sample.*

*Relevant scholarly references have been incorporated into the discussion section, providing a robust theoretical foundation for interpreting our findings.*
