# Supplementary material for: Behavioural activation activities for employees in the Chinese culture: A workshop
Source: PLOS Ment Health. 2024 Dec 20;1(7):e0000190. doi: 10.1371/journal.pmen.0000190 (PMC12798598; doi:10.1371/journal.pmen.0000190)
Supplement: Attachment — Submitted filename: Second Response to Reviewers.docx [file pmen.0000190.s006.docx]

Dear Editor and Reviewer,

We are very grateful to your comments and reviews. They have made our contribution stronger.

We have made the following modifications to the two suggestions:

1. Mention the exact type of mixed methods study they conducted and why they think it was the most appropriate

*The research methods are further explained: “This mixed-methods study combined quantitative methods, where participants' votes on each activity on the Miro board were recorded and statistically analyzed, with qualitative methods, capturing and thematically analyzing conversation content from their discussions. These methods were chosen because they facilitate an inclusive, collaborative environment that harnesses the diverse perspectives of participants, essential for culturally sensitive adaptations.”*

2. The added participant quotes under qualitative results which is great, however they don’t indicate the exact participants who made the statements. This is required practice in qualitative research to indicate source.

*The alias, gender, age, and occupation of the participants are added to the end of each quote.*

With regards

The authors
